# Non-fistulous Bladder Ulceration in Crohn's Disease: A Rare Extraintestinal Manifestation

**DOI:** 10.7759/cureus.92102

**Published:** 2025-09-11

**Authors:** Alaa Jazzar, Sani Beydoun, Mahmoud Hallal, Walid Alameh

**Affiliations:** 1 Urology, Lebanese University, Beirut, LBN; 2 Gastroenterology and Hepatology, Lebanese University, Beirut, LBN; 3 Urology, Sahel General Hospital, Fakhry Alame University Medical Center (FAUMC), Beirut, LBN

**Keywords:** auto immune, bladder, crohn's disease (cd), extraintestinal manifestations in inflammatory bowel disease, gross hematuria

## Abstract

Non-fistulous bladder ulceration is an extremely unusual extraintestinal manifestation of Crohn's disease (CD) with significant diagnostic and therapeutic challenges. We present a case of a 21-year-old male with ileocolonic CD, post-resection, who was admitted with repeated gross hematuria. Initial imaging (CT/MRI) revealed bladder wall thickening and an enterocolonic fistula, but no enterovesical communication. Cystoscopy revealed widespread mucosal erythema and ulceration, and histopathology was in keeping with chronic inflammation but not malignancy or granulomas. Multidisciplinary evaluation excluded malignancies and infections and led to a diagnosis of non-fistulous bladder ulceration associated with CD. Escalation of infliximab led to the resolution of hematuria and sustained remission at six-month follow-up. This case highlights the importance of recognition of atypical bladder involvement in CD patients with urinary symptoms, even in the absence of fistulas. Advanced imaging techniques, cystoscopy, and multidisciplinary cooperation are crucial for accurate diagnosis. Anti-tumor necrosis factor (TNF) therapy, particularly dose-intensified regimens, can effectively manage both intestinal and extraintestinal inflammation, suggesting the participation of shared immunopathogenic mechanisms. This review emphasizes the need for heightened clinical suspicion and multidisciplinary management to optimize outcomes in atypical CD-related complications.

## Introduction

Crohn's disease (CD), which is a chronic inflammatory bowel disease characterized by inflammation that is transmural, primarily affects the gastrointestinal tract but is also seen with a spectrum of extraintestinal manifestations (EIMs) and complications [1,2]. While fistulizing disease, particularly enterovesical fistulas, is a well-documented complication of CD, non-fistulous inflammation of the bladder is exceptionally rare [3,4]. This case report documents a rare case of isolated bladder ulceration in a patient with CD, unrelated to fistulization, and highlights the diagnostic and therapeutic challenges posed by this rare presentation. Complications of CD are usually strictures, abscesses, and fistulas, with enterovesical fistulas being seen in 2-10% of patients due to transmural inflammation and erosion into adjacent organs [3,5].

In contrast, non-fistulous bladder involvement is poorly understood and rarely documented. Until now, only anecdotal evidence, such as Limdi et al. [6] and Triantafillidis et al. [7], has described bladder ulceration in CD patients without fistulae. The pathogenesis of these lesions is not yet known, but may be a result of shared immunopathological mechanisms or direct inflammatory spread [8]. Histopathological study usually reveals chronic cystitis with granulomatous changes consistent with intestinal disease, though there are no agreed-upon diagnostic criteria. This case emphasizes the importance of considering abnormal bladder pathology in CD patients with urinary symptoms, even in the absence of fistulas. Early diagnosis and a multidisciplinary approach are needed to avoid morbidity and optimize outcomes [9]. 

## Case presentation

A 21-year-old male with a three-year history of ileocolonic CD (Montreal A2L3B1) presented with two months of recurrent gross hematuria. His vital signs were normal: blood pressure 120/75 mmHg, heart rate 78 bpm, temperature 36.8°C. Physical examination revealed mild lower abdominal tenderness.

Laboratory findings showed hemoglobin 12.7 g/dL, leukocytosis 12,300/uL. Fecal calprotectin was 170 μg/g (normal <50 μg/g). Urinalysis showed >100 RBCs/hpf, and the urine culture was negative. 

**Figure 1:**
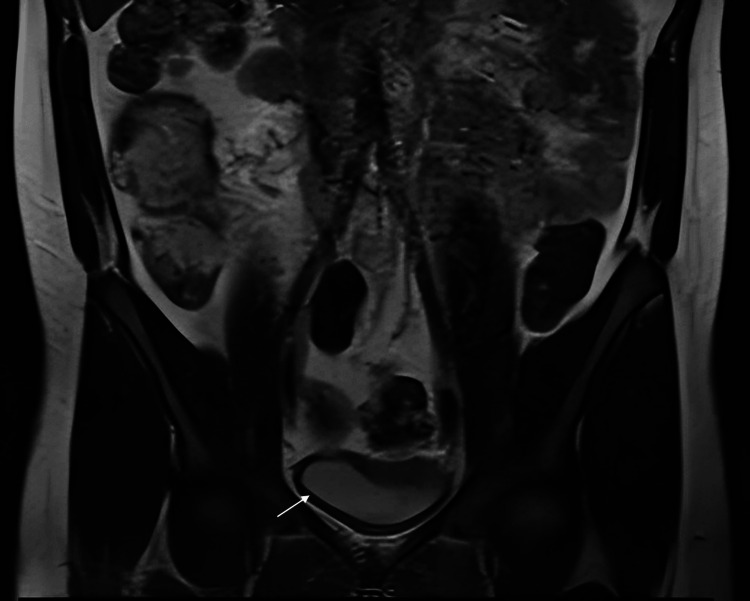
Coronal T2-weighted MRI of a patient with Crohn's disease, showing mild bladder wall thickening (arrow) and adjacent bowel changes suggestive of active disease, without evidence of an enterovesical fistula.

CT abdomen-pelvis showed only mild bladder wall thickening (6 mm). MRI of abdomen-pelvis with gadolinium showed a 2.7 cm enterocolonic fistula (terminal ileum to sigmoid colon) and reactive lymphadenopathy. There was no evidence of enterovesical fistula (Figure 1).

**Figure 2:**
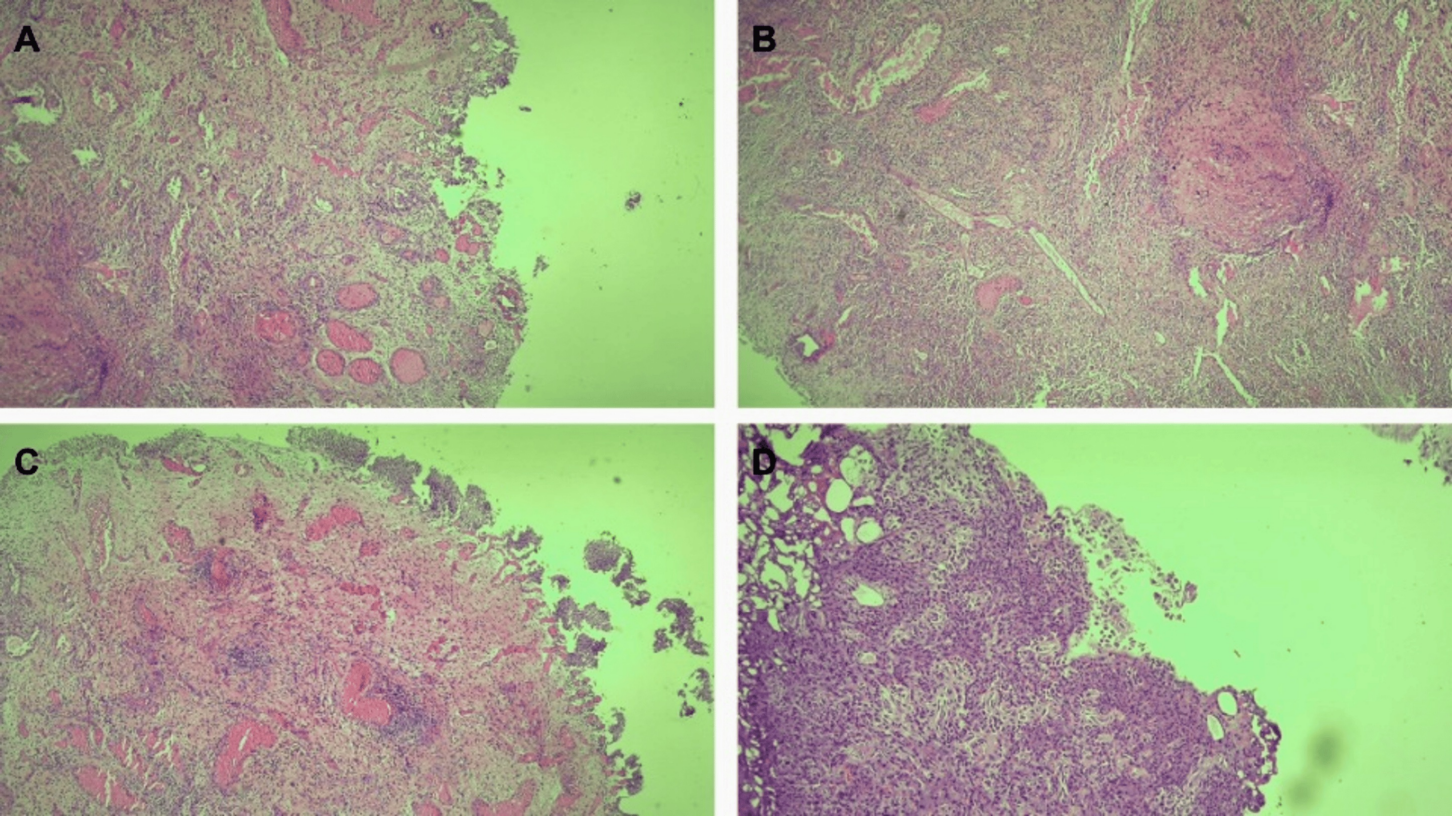
Urinary bladder biopsy specimen showing mucosal ulceration with severe acute and chronic inflammation and congestion in the lamina propria (A, D), focal granulomatous inflammation (B), and reactive urothelium (C) (H&E, ×20).

A cystoscopy was performed, which revealed a diffuse mucosal erythema and "tears of blood" along the posterior bladder wall and aspect of ulcerations. Biopsies revealed granulomatous ulceration and intense inflammation without malignancy (Figure 2).

Immunostains for CK20 and p53 were negative. After discussion with the gastroenterologist, it was decided to increase the infliximab dose to 10 mg/kg every four weeks. Pre-infusion screening excluded latent infections.

At six months, the patient had attained sustained remission, with resolution of gross and microscopic hematuria, which was documented by a negative urine analysis and CD flares. CD Biomarkers were normalized (C-reactive protein (CRP)<1 mg/dL and fecal calprotectin <50 μg), and the follow-up MRI showed significant improvement in the previously noted inflammatory bowel changes, with complete resolution of the bladder wall thickening. 

## Discussion

This case illustrates the diagnostic and therapeutic challenge of fistula-negative bladder ulceration in CD, a phenomenon that defies traditional notions of CD-associated extraintestinal manifestations (EIMs). Gross hematuria, a sudden and striking finding in this case, contrasts with the more typical fistulizing complications of CD, which are most often in the form of pneumaturia or recurrent urinary tract infections [4]. Hematuria has been rarely reported in CD-associated bladder involvement, but its presentation in this context without evidence of enterovesical fistula emphasizes the diagnostic dilemma of non-fistulous inflammatory processes. Fistulizing complications such as enterovesical fistulas are typically well-described [3,5]. However, bladder inflammation without fistula development remains a diagnostic challenge. The absence of an enterocolonic fistula in our case also highlights the multifactorial nature of CD-associated inflammation, in which transmural disease activity can cause both fistulous and non-fistulous pathology through different mechanisms. The pathogenesis of non-fistulous bladder involvement in CD is founded on shared immunopathological mechanisms.

CD is driven by dysregulated Th1/Th17 immune responses with tumor necrosis factor (TNF-α) as the central mediator of inflammation [1,10]. Overexpression of TNF-α has been linked with both extraintestinal and intestinal inflammation, even in urogenital tissues, by enhancing the leukocyte recruitment and damaging epithelial barriers [11]. Resolution of our patient's bladder symptoms with the escalation of infliximab is consistent with this paradigm, implying that blockade of systemic TNF-α reduces inflammation throughout the various systems [9]. However, the absence of granulomas from bladder biopsies contrasts with previous reports of granulomatous cystitis [6] and emphasizes the phenotypic heterogeneity of CD-associated bladder disease. This heterogeneity may reflect differences in local cytokine environments or genetic susceptibility, as polymorphisms within the NOD2/CARD15 susceptibility gene for CD have been linked with severe EIMs [12]. Bladder disease not associated with a fistula is often a diagnosis of exclusion in CD. Early modalities such as CT are not sensitive for the detection of subclinical transmural inflammation or early fistulas [3].

In our patient, MRI with gadolinium enhancement played a key role in identifying the enterocolonic fistula and bladder wall edema not appreciated on CT. MRI superiority in characterizing CD complications is well established, with Rimola et al. [13] meta-analysis finding 85-90% accuracy for fistula detection compared to 50-70% for CT. Diffusion-weighted imaging (DWI) sequences add to the visualization of inflammatory activity without contrast [14]. Cystoscopy and biopsy are nevertheless needed for exclusion of malignancy or infection, even if histopathological findings (e.g., ulceration, chronic inflammation) are non-specific and may resemble those of other entities such as interstitial cystitis [15]. The success of anti-TNF therapy in this case is consistent with growing evidence for the use of TNF-α inhibitors in refractory EIMs. A multicenter cohort study by Peyrin-Biroulet et al. [11] had 70% efficacy of infliximab in the healing of EIMs, including uveitis and pyoderma gangrenosum, at 12 weeks.

Notably, bladder inflammation is expected to improve before fistulizing disease due to differing underlying mechanisms (e.g., TNF-mediated mucosal vs. fibrotic pathways) [16].

The optimal dosing regimen for non-fistulous EIMs remains to be established. Increase to 10 mg/kg q4 weeks, employed here, mimics strategies for penetration through CD and perhaps enhances drug infiltration into inflamed tissues [17]. Multidisciplinary collaboration was the focus of this case, and one that concurs with advice that emphasizes coordinated care for complex CD [2,9]. Urologic specialty-guided cystoscopy and biopsy interpretation, and radiologists adjusted MRI protocols to detect latent fistulas. This kind of collaboration is particularly crucial when it comes to differentiating CD-associated cystitis from coexisting diseases like bladder cancer or infection that require different treatment [18]. This case is limited by its single-patient design and limited follow-up. Larger trials need to ascertain the frequency of non-fistulous bladder involvement in CD, which recent papers estimate at <1% [6,7]. Prospective registries, such as the European Crohn's and Colitis Organisation (ECCO) EIMs database, would clarify risk factors and natural history [19]. Additionally, translational research on bladder-specific biomarkers (e.g., urinary TNF-α, calprotectin) would facilitate non-invasive diagnosis [20].

## Conclusions

Non-fistulous bladder ulceration is a rare complication of CD that should be considered in patients with unexplained urinary symptoms. Accurate diagnosis requires advanced imaging, cystoscopy, and a multidisciplinary approach. This case demonstrates that escalation of anti-TNF therapy can effectively manage both intestinal and urinary manifestations.
